# Ironing out the wrinkles in the rare biosphere through improved OTU clustering

**DOI:** 10.1111/j.1462-2920.2010.02193.x

**Published:** 2010-07

**Authors:** Susan M Huse, David Mark Welch, Hilary G Morrison, Mitchell L Sogin

**Affiliations:** Josephine Bay Paul Center, Marine Biological Laboratory at Woods Hole7 MBL Street, Woods Hole, MA 02543, USA

## Abstract

Deep sequencing of PCR amplicon libraries facilitates the detection of low-abundance populations in environmental DNA surveys of complex microbial communities. At the same time, deep sequencing can lead to overestimates of microbial diversity through the generation of low-frequency, error-prone reads. Even with sequencing error rates below 0.005 per nucleotide position, the common method of generating operational taxonomic units (OTUs) by multiple sequence alignment and complete-linkage clustering significantly increases the number of predicted OTUs and inflates richness estimates. We show that a 2% single-linkage preclustering methodology followed by an average-linkage clustering based on pairwise alignments more accurately predicts expected OTUs in both single and pooled template preparations of known taxonomic composition. This new clustering method can reduce the OTU richness in environmental samples by as much as 30–60% but does not reduce the fraction of OTUs in long-tailed rank abundance curves that defines the rare biosphere.

## Introduction

Massively parallel pyrosequencing of ribosomal RNA (rRNA) coding regions that evolve rapidly allows the detection of very low abundance populations in complex microbial communities. Each processed sequence (pyrotag) more or less serves as a proxy for the occurrence of a microbial genome in an environmental DNA sample. Matching pyrotags to a reference rRNA database or clustering tags in a taxon-independent manner to identify operational taxonomic units (OTUs) suggests that taxonomic richness in marine, terrestrial and microbiome communities exceeds all prior estimates of microbial diversity. Many of the novel OTUs correspond to low-abundance organisms of the ‘rare biosphere’ (Pedros-Alio, 2006; 2007;). However, the ability to generate more comprehensive descriptions of microbial diversity through deep sequencing introduces new challenges when estimating diversity, and questions have been raised about the accuracy of OTU richness estimates and the extent of the rare biosphere. Sequencing errors coupled with inadequate choice of clustering algorithms can lead to artificially elevated estimates of community richness.

Quality-controlled sequence reads of 16S rRNA V6 hypervariable region amplicon libraries produced by the Roche Genome Sequencer 20 System (GS 20) have a per-base error rate of 0.25% (Huse *et al.*, 2007), which is comparable to a average phred score better than 25 on contemporary capillary instruments (Ewing and Green, 1998; Ewing *et al.*, 1998). For targets such as the short V6 rRNA hypervariable region that initially revealed the existence of the rare biosphere, 1 inaccurate nucleotide per 400 positions translates into 13% of the reads containing at least 1 inaccuracy. The number of sequences containing errors scales with the size of the sequencing effort, with larger data sets yielding a greater number of distinct sequencing variants. Instead of estimating diversity according to the number of unique sequences recovered, clustering methods based on either phylogenetic inferences or sequence similarities can define membership in an OTU. Similarity between rRNA genes equal to or greater than 97% often serves as a benchmark for assigning sequences to the same OTU. Yet even with very low sequencing error rates, the very large data sets produced by massively parallel sequencing will inevitably contain a fraction of reads with multiple errors, which can lead to overestimates of diversity.

Recent reports have demonstrated the difficulties in clustering sequences into the appropriate number of OTUs in a sample based upon either unique rRNA pyrotags or 3% clusters. Kunin and colleagues (2010) explored how intrinsic pyrosequencing errors can lead to inflated estimates of diversity. Using the Markov Cluster algorithm (Van Dongen, 2008) they identified several hundred OTUs in pairwise alignments of ∼8000 300 nt reads from both the 5′ (4254 reads) and the 3′ end (4244 reads) of the *Escherichia coli* MG1655 rRNA gene. They examined the impact of quality filters including those outlined by Huse and colleagues (2007) as well as filters thatincorporate an improved quality-scoring algorithm that allows for a minimum average quality score and more stringent end-trimming (Brockman *et al.*, 2008). By applying a stringent quality filter minimum of ≤ 0.2% error and computing 3% pyrotag clusters, Kunin and colleagues (2010) obtained the predicted estimates of diversity for a single microbial genome.

A different strategy for minimizing the contribution of pyrosequencing error to inflated estimates of diversity relies upon the program PyroNoise (Quince *et al.*, 2009). This algorithm corrects pyrosequences that are statistically more likely to be a variant of a common read caused by pyrosequencing error than an infrequent novel sequence. Rather than relying on the Roche base-calling algorithm and associated quality scores, PyroNoise evaluates the underlying flowgrams from the Roche Genome Sequencer FLX System (GS FLX). The combination of PyroNoise, multiple sequence alignment and complete linkage clustering (MS-CL) identified ∼60 OTUs in an analysis of pyrotags from a pooled template preparation (Quince *et al.*, 2009). Without PyroNoise, the same clustering procedure inflated diversity to ∼6000 OTUs. In an environmental sample of 16 222 pyrotags from Priest Pot Lake, PyroNoise reduced the OTU richness from 1327 to 855. However, the current implementation of PyroNoise requires very large computational resources (Quince *et al.*, 2009).

Sun and colleagues (2009) evaluated the impact of using pairwise (PW) alignments between sequences rather than the commonly used multiple sequence (MS) aligners, e.g. MUSCLE (Edgar, 2004), ClustalW (Thompson *et al.*, 1994) or NAST (Desantis *et al.*, 2006). Pairwise alignments reduced the number of OTUs by ∼30% compared with MS alignments. Performing pairwise alignments for hundreds of thousands of sequences requires a large number of CPU cycles. Sun and colleagues (2009) devised a filter using the more efficient kmer distances to preselect from all possible pairwise combinations only those combinations needed for clustering. For example, precisely computing pairwise distances for sequences differing by more than 10% is not required for creating accurate 3% clusters. Their program ESPRIT incorporates the kmer prefiltering of pairwise distances followed by a Needleman-Wunsch alignment (Needleman and Wunsch, 1970) for all sequence pairs of interest. This program makes pairwise alignments feasible for even very large samples.

Each of these studies focused on one aspect of determining OTUs based on sequence similarity, either pyrosequencing error or alignment. Here we evaluate multiple facets of the entire clustering process, specifically the contribution of sequencing error, alignment method and clustering algorithm to the number of observed OTUs for communities of known taxonomic composition. We set our cluster threshold at 3% to minimize the influence of sequencing errors and to be consistent with the work cited above and with the majority of microbial diversity publications (Stackebrandt and Goebel, 1994). We amplified and pyrosequenced the V6 hypervariable region of the ribosomal RNA gene from single- and multiple-template pools to determine a simple and computationally effective method for accurately clustering short hypervariable pyrotags. We then applied this new clustering method to multiple published environmental samples to determine the nature and extent of OTU inflation in diverse samples.

## Results

To assess the accuracy of sequence clustering methods, we generated amplicon libraries of the V6 region from several DNA preparations of known 16S rRNA coding region composition: (i) cultures of a single clone (colony forming unit) of *E. coli*, which contains seven 16S operons with two distinct V6 hypervariable region sequences; (ii) cultures of a single clone of *Staphylococcus epidermidis*, which contains six 16S rRNA operons with two distinct V6 sequences; (iii) a plasmid clone containing a nearly full-length 16S rRNA coding region from *E. coli* 16S rRNA operon A, (iv) a plasmid clone containing a nearly full-length 16S rRNA coding region from *S. epidermidis* 16S rRNA operon 9; and (v) a mixture of 43 plasmid clones (Clone-43) containing partial 16S rRNA coding regions that span the V6 hypervariable region of distinct 16S rRNA genes amplified from a deep-sea hydrothermal vent community (Huse *et al.*, 2007). We sequenced approximately 200 000 high-quality reads from each of the genomic template *E. coli* and *S. epidermidis* libraries and from the mixed template Clone-43 library, and approximately 30 000 high-quality reads from the single template *E. coli* and *S. epidermidis* plasmid clone libraries. The amplified V6 regions of *E. coli* and *S. epidermidis* are 60 and 62 nt respectively. The V6 region of the Clone-43 mixture ranges from 57 to 145 nt.

### PCR and pyrosequencing error

Prior to calculating the sequencing error rate, we eliminated the 11–19% of all reads that did not pass previously described quality filtering (Huse *et al.*, 2007; Kunin *et al.*, 2010). Our requirement that valid reads and their best match in a database of 16S rRNA sequences have an alignment ≥ 80% of the read's length (Table S1) eliminated an additional 0.02–0.14% of the reads. Comparing the reads that failed this criterion to the GenBank, non-redundant sequence database showed that many represented non-V6 amplification products. A small number of the aberrant reads contained insertion artefacts in the V6 amplicons. Short, highly diverse amplicon libraries that do not contain conserved regions will produce few if any chimeric reads (S.M. Huse, unpubl. obs.), and we assume that the minimum GAST alignment length requirement removed any inter-template chimeras in the Clone-43 data set. The large number of similar amplicons in the *E. coli* and *S. epidermidis* genomic template data sets led to the formation of several obvious within-species chimeras that we identified and removed based upon our knowledge of the sequences.

Differences between a pyrotag sequence and its template reflect a combination of PCR and pyrosequencing error. We calculated the combined error rate, understanding that any unfiltered contamination, chimeras or non-rRNA amplification will increase our error rate estimates. We define the error rate for a given sequence as the number of insertions, deletions and substitutions in a sequence as compared with its template, divided by the length of the template sequence. The error rate varied between data sets, ranging from 0.0021 to 0.0042 (Table S2). This fits within the range we reported previously using a GS20 platform (Huse *et al.*, 2007) and compares with the error rate of capillary sequence data with an average phred score of 24-27.

If pyrosequencing errors occurred at random, the number of expected sequences with exactly *n* errors would follow the binomial distribution. Figure S1 shows that sequences having 1 error occurred less frequently than expected, while those with 3 or more errors exceeded expectations for a random distribution. Thus, a small proportion of the sequences contained multiple errors not predicted by the binomial distribution. In analyses of GS20 data, we previously reported that a limited number of sequences contained multiple errors (Huse *et al.*, 2007), which agrees with the known propensity for pyrosequencing technology to produce a small number of reads with many errors likely from beads on which the growing DNA strands fall out of phase or from multi-templated beads of similar sequence. Despite this non-random distribution, the vast majority of reads (> 99%) display no more than two differences (< 3.5% variation) from their parent templates (Table S2). We had previously assumed that pyrotags with 3% or fewer errors would cluster with their parent sequence while errant pyrotags containing more than 3% errors would likely form a new singleton OTU, but might occasionally join a cluster of closely related errant sequences. Table 1 shows that the number of 3% OTUs calculated using the common method of multiple sequence alignment and complete-linkage clustering (MS-CL) exceeds the sum of expected template OTUs (based upon the known number of rRNA coding regions in each template pool) plus the number of clusters that would occur if each errant sequence > 3% variant formed a new OTU. This suggests that sources other than pyrosequencing error can inflate OTU estimates.

**Table 1:** Number of OTUs for different clustering methods.

	Expected OTUs	Maximum expected due to errors	MS-CL	PW-AL	SLP/PW-AL
Template samples					
*E. coli* (*n* = 215 618)	2	378^1^	1042	277	88
*E. coli* SSU A (*n* = 31 030)	1	23^1^	137	25	10
*S. epidermidis* (*n* = 197 876)	2	769^1^	1267	323	128
*S. epidermidis SSU 9* (*n* = 37 587)	1	85^1^	205	37	20
Clone-43 (v6) (*n* = 202 340)	43	630^1^	2473	458	275
Clone-43 v4-5 (232 nt, *n* = 16 673)	42	23^1^	126	51	54
Clone-90 (241 nt, *n* = 34 308)	30	34–69^2^	237	65	62
Natural samples					
Deep-sea vent *Archaea* (*n* = 63 133)	N/A	63–126^2^	709	483	470
English Channel (*n* = 12 851)	N/A	13–26^2^	1154	880	859
Human Gut (*n* = 15 239)	N/A	15–30^2^	803	625	566
Sewage (*n* = 33 082)	N/A	33–66^2^	2383	1881	1831
North Atlantic Deep Water (*n* = 15 497)	N/A	15–30^2^	1713	1363	1339

For both known template and environmental samples, we calculated the number of expected OTUs (known templates only) and the number generated using several alignment and clustering methods. We calculated the maximum additional OTUs expected due to errors using either (1) the count of unique sequences having more than 2 errors in the template pool (superscript ‘1’ in the table), or (2) 1–2 OTUs for every 1000 tags (superscript ‘2’ in the table). MS is a multiple sequence alignment, PW is a pairwise alignment, CL is complete-linkage clustering, AL is average-linkage clustering, and SLP is single-linkage preclustering.

### Effect of alignment and clustering methods

To evaluate the tendency of clustering methods to inflate the number of OTUs, we examined the behaviour of various approaches on sequences that differed by less than 3.5% (or 2 errors) from their template [the commonly used program, mothur (Schloss *et al.*, 2009), rounds distances to the nearest whole per cent when defining a cluster]. We found that MS-CL returned 129 instead of the two expected *E. coli* OTUs, 89 rather than the two expected *S. epidermidis* OTUs and 694 Clone-43 OTUs rather than the expected 43 (Table 2). Clearly, the clustering methodology propagates additional OTUs. To determine the cause(s) of this inflation we examined the effects of sequence alignment and clustering method on the number of OTUs generated from sequences that differ by less than 3.5%.

**Table 2:** Effect of clustering algorithm on sequences within 3% of their template.

	*Escherichia coli*	*Staphylococcus epidermidis*	*Escherichia coli* operon A	*Staphylococcus epidermidis* operon 9	Clone-43
Expected	2	2	1	1	43
MS-CL	129	89	29	26	694
MS-AL	54	44	6	12	218
MS-SL	2	2	1	2	57
PW-CL	6	5	1	1	308
PW-AL	2	2	1	1	43
PW-SL	2	1	1	1	43

For each of five defined template preparations, we used combinations of alignment and clustering methods to create 3% OTUs.

We first tested the effect of alignment strategies on the number of OTUs. Differences between hypervariable rRNA coding regions include a combination of elevated rates of nucleotide substitution, insertions and deletions that lead to considerable uncertainty in multiple sequence alignments. Sun and colleagues (2009) showed that combining thousands of such diverse sequences in one alignment can inflate the number of OTUs. Their pairwise alignments of pyrotags consistently yielded fewer OTUs than the multiple sequence alignments from MUSCLE, NAST or RDP-Pyro. We found that using a Needleman-Wunsch pairwise alignment rather than an unstructured multiple sequence alignment removed from 56% to 97% of the spurious OTUs in data sets containing only sequences less than 3.49% different from their template (Table 2).

We examined the effect of three clustering algorithms: complete linkage, i.e. furthest neighbour, average linkage, i.e. average neighbour or upgma and single linkage, i.e. nearest neighbour. These algorithms employ different rules to determine whether a new sequence adds to an existing cluster or initiates formation of a new cluster. When using a 3% cut-off criterion for adding sequences to a cluster, complete linkage requires that a new sequence be less than 3% different from *every* other sequence already in the cluster; average linkage requires that the *average* distance between the new sequence and every sequence already in the cluster be less than 3%; and single linkage requires only that the new sequence be less than 3% different from *any one* read within the cluster. Regardless of alignment method, complete linkage produced more OTUs than expected (Table 2).

**Fig. 1:**
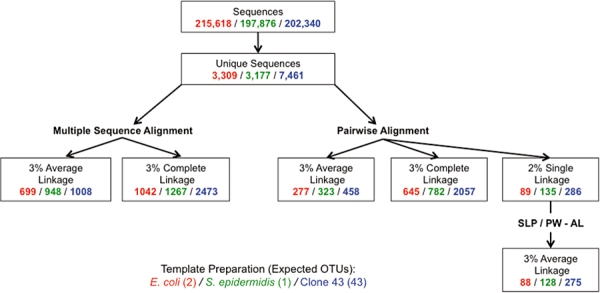
Effect of clustering method on the number of OTUs. We created OTU clusters of the three known template preparations using combinations of multiple sequence and pairwise alignments, complete-linkage and average-linkage clustering, and single-linkage preclustering. Each method provides distinctly different numbers of OTUs for the same data. For short hypervariable tags sequenced at depth, the single-linkage preclustering using pairwise alignments, followed by an average linkage clustering (SLP / PW-AL) provides the most accurate results.

In the absence of sequencing error greater than 3%, the combination of pairwise alignment and average-linkage clustering successfully eliminated all spurious OTUs from our five simplified data sets containing errors < 3% (Table 2). Quince and colleagues (2009) also found that average-linkage clustering provides better results in the presence of noise. We applied this method to complete data sets (containing errors > 3%). As expected, we eliminated most but not all spurious OTUs because the data sets include sequences with too many errors to cluster with their template (Table 1, Fig. 1).

### Creating preclusters represented by single sequences

To reduce the number of spurious clusters produced by errant sequences, we introduced a modified single-linkage preclustering (SLP) step. We sorted sequences in abundance order before using the single-linkage algorithm to cluster sequences that differ by only 2%. Unlike other single-linkage algorithms, SLP does not merge two clusters when incorporating a new sequence that is within 2% of both; instead, it assigns the new sequence to the cluster containing the most abundant sequences. We chose a 2% preclustering width because the preclustering width must be less than the final clustering width, and because 2%, which corresponds to a single nucleotide change in a short, 60 nt read, is the smallest threshold that can be applied to V6 pyrotag sequences. Finally, SLP assigns the most abundant sequence as the representative of each precluster in a 3% average-linkage clustering of the full data set. This single-linkage preclustering approach reduced the number of spurious OTUs in data sets of known composition by ∼90% (Table 1). We applied this method to the analysis of longer pyrotags from DNA preparations of known composition: the V4-V5 region of Clone-43 (average sequence length 232 nt) and a data set of ∼240 nt including the V5 region from 90 plasmid templates (Clone-90) initially analysed by Quince and colleagues (2009). These longer sequences show lower rates of OTU inflation, perhaps because so many more changes in the sequence are needed to exceed 3%. The SLP step removed nearly all of the spurious OTUs from both of these data sets (Table 1). Single-linkage clustering normally carries the risk of over-aggregating sequences in complex samples. If the sequence space contains abundant template sequences surrounded by decreasing numbers of variants, then presorting by abundance prohibits the chaining together of template sequences with increasing divergence. Only those templates that are less than 3% different will fall into the same cluster, and these sequences should cluster together under any algorithm. Inspection of the OTUs generated in all of these samples showed that the SLP method succeeded in placing variant sequences with three or even four errors into OTUs with their template sequence, but did not aggregate template sequences that differed by more than 3%.

**Fig. 2:**
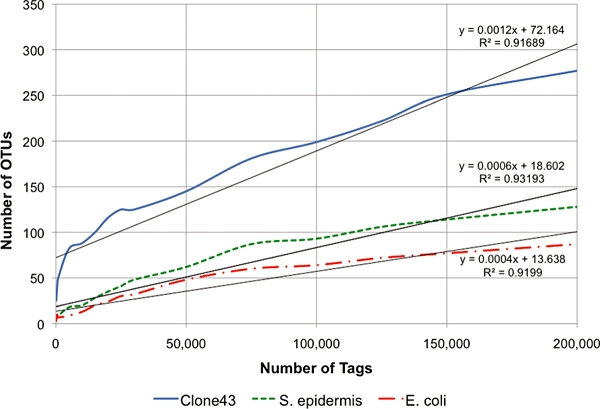
Number of additional OTUs as a function of sample depth. For the two genomic templates, *E. coli* and *S. epidermidis*, and the multiple template Clone-43 samples, we calculated the number of spurious OTUs as a function of sample depth.

To assess the remaining OTU inflation caused by errant sequences that SLP did not aggregate, we used SLP/PW-AL to determine OTUs in subsamples of the two genomic templates and the multiple-template Clone-43 data set at depths from 100 to ∼200 000 tags (197 846 sequences for *S. epidermidis*). As Fig. 2 illustrates, the number of spurious OTUs generated is a function of sampling depth. The Clone-43 sample, with the highest ratio of spurious OTUs, has 234 extra OTUs at a sampling depth of 200 000. The *E. coli* sample had a rate less than half of that with only 85 extra OTUs at 200 000 sequences. Although the curves are not linear, they show that we can expect between one and two spurious OTUs per 1000 reads.

### Comparison to PyroNoise

We compared the SLP approach to PyroNoise (Table S3), using two samples previously analysed with PyroNoise (Quince *et al.*, 2009), and three additional samples (two environmental and the Clone-43 pooled-template preparation). Both methods reduced the number of OTUs by approximately 30–50%. None of the SLP/PW-AN OTUs contained multiple high abundance sequences that differed by more than 3%.

### Impact on environmental data sets and the rare biosphere

We compared the effect of the MS-CL and SLP/PW-AL methods of clustering on the distribution of OTU abundances in bacterial V6 amplicon libraries representing samples from the English Channel (Gilbert *et al.*, 2009), Milwaukee sewage (McLellan *et al.*, 2010), the human gut (Turnbaugh *et al.*, 2009), the North Atlantic Deep Water (Sogin *et al.*, 2006) and an archaeal V6 amplicon library from a deep-sea hydrothermal vent (Huber *et al.*, 2007). SLP/PW-AL generated 30–40% fewer OTUs than MS-CL (or SLP/MS-CL, Table S4). The relative frequency of the most abundant OTUs was 10–50% greater using SLP/PW-AN, consistent with the aggregation of errant sequences with their template that occurs during the SLP step (Table 3). Neither the fraction of OTUs that contained only a single tag (singleton OTUs) nor the fraction of OTUs that contained 1–3 tags differed substantially between methods. Thus, while SLP/PW-AN reduces the total number of OTUs it does so across a wide range of OTU abundances and does not reduce the proportion of OTUs that comprise the long tail of the abundance curve.

**Table 3:** Distribution of most and least abundant pyrotags in MS-CL and SLP/PW-AL clusters.

	Deep-sea vent *Archaea*	English Channel	Human Gut	Sewage	North Atlantic Deep Water
Size of most abundant OTU MS-CL	47 711	1613	836	948	1371
Size of most abundant OTU SLP/PW-AL	49 857	1882	1436	4219	1711
Total OTUs MS-CL	709	1154	803	2383	1713
Total OTUs SLP/PW-AL	470	859	566	1831	1339
Per cent OTUs as singletons – tripletons MS-CL	39%	66%	65%	69%	74%
Per cent OTUs as singletons – tripletons SLP/PW-AL	42%	64%	64%	69%	77%

## Discussion

The increased molecular sampling effort enabled by massively parallel DNA sequencing allows the detection of very-low-abundance DNA molecules in complex PCR amplicon libraries. In diversity surveys, these low-abundance sequences delineate rare OTUs (containing one or a few sequence tags) that define the long tail of rank abundance curves for complex microbial communities. This distribution implies the existence of a rare biosphere of many hundreds or thousands of very low abundance phylotypes. The extent of the long tail could reflect true biological signals of diversity, or deep molecular sampling efforts might amplify the effect of sequencing errors and clustering methods when estimating OTU richness. In their review of techniques for minimizing pyrosequencing error, Reeder and Knight (2009) suggested that the majority of pyrotags that make up the rare biosphere represents the accumulation of small sequencing errors. By extrapolating the rate of OTU inflation observed for low-complexity amplicon libraries, e.g. templates containing rRNA genes for either a single taxon or 90 taxa, they predicted similar impacts on diversity estimates for complex microbial communities and that the reduction in OTUs would correspond to rare biosphere populations. To determine how sequencing error might inflate estimates of OTUs, we undertook an in-depth analysis of template pools of known rRNA composition to determine the contribution of sequencing error to estimates of microbial diversity, the effect of alignment and clustering methodology when defining OTUs from massively parallel pyrotag data sets, and the contribution of aberrant amplicons templated from non-rRNA coding regions. After filtering low-quality sequences, the per-base pyrosequencing error rate on the GS FLX platform did not exceed conventional capillary systems. However, the behaviour of common alignment and clustering algorithms led to significant inflation of 3% OTUs commonly cited in studies of microbial diversity. Very large data sets irrespective of sequencing technology exacerbate the influence of these factors on estimates of diversity.

Before estimating the number of OTUs predicted by pyrosequencing of the pooled template preparations, we removed low-quality reads and identified sequences that did not represent targeted hypervariable regions. In the case of DNA extracted from *E. coli* and *S. epidermidis* cultures, many of the non-target reads mapped to specific regions of the genome that did not code for rRNAs. In some genomes our rRNA primers will bind with low efficiency to similar but non-identical targets to produce non-rRNA pyrotags. This contamination by non-rRNA amplicons may vary for different taxa and may increase for complex communities with greater aggregate genetic complexity. It also reinforces the importance of screening reads for the inclusion of both primers, a process that is more difficult when pyrosequencing read lengths fail to span longer amplicon targets. While the fraction of these reads may be low, they contribute disproportionately to the creation of spurious OTUs; most represent unique sequences that will form novel clusters at any level. Reliance upon a significant local match by blast fails to identify these reads since the local match can be quite short. Requiring the alignment to extend for at least 80% of the length of the read effectively filters these non-target sequences from the pyrotag data set. A minimum alignment length can also detect chimeras between divergent taxa, although short hypervariable tags from diverse communities appear to generate few if any chimeras (S.M. Huse, unpublished analysis of 25 million pyrotags).

After removing low-quality sequences (Huse *et al.*, 2007; Kunin *et al.*, 2010) and reads that did not represent the targeted region, we measured the per-base error rate to be 0.0021–0.0042. (We also identified a small number of V6 rRNA reads from microbial taxa that we did not intentionally include in the initial DNA preparations. An examination of sterile nutrient broth revealed that the yeast extract component contained DNA from diverse microbial taxa, data not shown.) This represents a combination of *in vivo* DNA replication errors, PCR errors and incorrect base calls by the Roche GS FLX software. The distribution of errors did not follow a binomial distribution, as we would expect if errors were randomly distributed in an independent manner throughout the reads. The measured error rate of ∼0.003 failed to account for the number of 3% OTUs produced using MS-CL, because sequences with less than 3% errors did not form a single cluster with their parent sequence. The inability to optimize in a single alignment a large number of hypervariable sequences from highly divergent taxa exacerbates the difficulty of clustering errant sequences with their parent sequence. The presence of multiple OTUs that should cluster together at the 3% level may explain most of the OTU inflation.

The use of the complete-linkage clustering algorithm also inflates the number of estimated OTUs: as the amount of variation within the limited sequence space increases, it is decreasingly likely that variants will meet the requirement that no sequence can be added to an OTU unless it is less than 3% different from every sequence already in the OTU. Reads that vary from their template at different locations may be within 3% of the template, but not within 3% of each other. Each subsequent variant can initiate the formation of a new OTU. This leads to many individual OTUs comprised of variants less than 3% from their parent sequence. We tested clustering methods on mixed template preparations from which we filtered out variation greater than 3%, and demonstrated that clustering using the common method of an unstructured multiple sequence alignment followed by complete-linkage clustering (MS-CL) grossly overestimates the number of OTUs, while a pairwise alignment and average-linkage clustering (PW-AL) accurately identifies a single OTU for each template.

To further reduce the inflation of OTUs even in data sets containing errant sequences, we developed a single-linkage preclustering method, SLP. The method assumes that template sequences will occur more frequently than their error-induced variants. We first sort sequences in order of abundance, and then create 2% single-linkage clusters. In a dense sequence space, single-linkage clustering can potentially chain together sequences so that OTUs can contain highly divergent but clearly related sequences. By starting with the most abundant sequences, we seed each cluster with template sequences and then add the less abundant variants; this prevents less frequent variants from linking templates that differ by more than 3% into the same OTU. We use the very narrow preclustering width of 2% to further reduce the likelihood of over-zealous aggregation, while still aggregating error-induced variation (the optimal width may vary with tag length). Careful examination of all clusters generated for both our mixed template data sets and the natural communities revealed no cases where an OTU contained abundant sequences that were not within 3% of each other.

Both our SLP method and PyroNoise map infrequent sequencing variants to more frequent sequences, and both appear to be effective in reducing the sequencing noise that can contribute to OTU inflation. SLP, however, can also map variations arising from PCR error and microevolution to representative sequences. SLP and PyroNoise provide comparable reductions in spurious OTUs (Table S3). The two methods are not mutually exclusive however, and could be used in tandem, first cleaning pyrosequencing error with PyroNoise then combining moderate variation with SLP. The computational expense of running SLP, however, is much less than the current version of PyroNoise.

Any sequencing method will have an intrinsic error rate, and given a large enough sample size the errant sequences will potentially inflate estimates of diversity. Empirically, we find that after SLP and average-linkage clustering, a smaller number of spurious OTUs remain than with other clustering methods. The majority of these remaining OTUs contain a single read, and we measure the rate of their occurrence in our defined V6 template pools to be ∼0.0015 per template. Thus, a data set of 1000 reads (a sampling effort common for Sanger sequencing) would have only 1–2 reads with enough errors to cluster independently of their parent sequence. However, a deeper survey of 10 000 or more reads made possible by next-generation technologies such as pyrosequencing will produce 10–20 additional reads. This is substantially less than the number of singleton OTUs found in most environmental data sets.

We examined the OTUs generated by SLP/PW-AL from each of our mixed template preparations and found that > 90% of the spurious OTUs contained only a single sequence; the rest generally contained two or rarely three. While indiscriminately removing all singleton OTUs will certainly reduce spurious OTUs, singleton OTUs frequently represent valid rare phylotypes in diverse environmental samples. We compared singleton OTUs from replicate single genome templates and found that even with very deep sequencing (> 60 000 reads/replicate), singletons from one replicate rarely occurred in other replicates. If parallel generation of the same singleton OTU rarely occurs at these depths, we argue that the same spurious OTU will rarely arise in multiple environmental samples. In a preliminary analysis using 487 samples from the International Census of Marine Microbes (ICoMM, http://icomm.mbl.edu), we found that the probability of an OTU found in one sample occurring in another sample was about the same for singleton OTUs and non-singleton OTUs (not shown).

Extrapolating OTU inflation from results of single template preparations can lead to unwarranted conclusions since the OTU inflation is a function of the sampling effort, not the sample diversity. For example, if a sample is sequenced to a depth of 20 000 tags, it will produce 20–40 spurious OTUs at the 3% threshold using SLP/PW-AN clustering. If the sample contains a single template, these 40 extra OTUs will constitute an inflation of 400%. In a sample with 100 different templates these OTUs would represent an inflation of 40%. In a complex environmental sample with more than 1000 phylotypes, the spurious OTUs would inflate estimates of diversity by less than 4%. Consistent with this prediction, SLP/PW-AL reduced the OTU counts in our simple template preparations by ∼90%, but OTU counts only decreased by 30–60% in complex environmental samples, a significant change but not different by orders of magnitude. Quince and colleagues (2009) reported very similar results with an 80% decrease in the OTUs of an artificial community, but only a 40% decrease in an environmental sample. Theoretically, when the sample diversity exceeds 1–2% of the sample depth, the OTU inflation (assuming 1–2 OTUs/1000 reads) will be less than 10%. When the sample diversity exceeds 5–10% of the sample depth, the OTU inflation will be less than 2%.

Our initial expectation was that sequencing errors and clustering artefacts would disproportionately affect the rare OTUs. As the new SLP/PW-AL method maps errant sequences to their template sequence, the more abundant OTUs tend to increase in number. The majority of these sequences, however, previously clustered into non-singleton OTUs. Although the SLP/PW-AL method removed many spurious OTUs, the shape of the OTU abundance distribution curve remains essentially the same – a small number of highly abundant species and a long tail of rare species.

## Experimental procedures

### Generation of amplicon libraries and pyrosequencing

We inoculated 5 ml of filter-sterilized nutrient broth with a single colony of *E. coli* K12 ATCC 10798 or *S. epidermidis* ATCC 14990, and grew these cultures overnight at 37°C to an OD_600_ of ∼1 [nutrient broth is 8 g Difco nutrient broth powder (Invitrogen) in Milli-Q+ ultrapure water (Millipore) to 1 l; all loops, pipets, flasks, etc. were disposable sterile polycarbonate or polypropylene]. For each species, we inoculated three 250 ml flasks, each containing 50 ml nutrient broth, with 100 µl of the overnight culture. We incubated the flasks at 37°C with vigorous shaking and harvested the cultures at late stationary phase (OD_600_ of ∼1.25). We then divided each culture into three aliquots, extracted genomic DNA and used each DNA extraction in a separate amplification of the V6 region. We split one amplicon library from each species into two emPCR amplification prior to pyrosequencing for a total of 10 subsamples of single clones of *E. coli* and of *S. epidermidis*. We also generated an amplicon library from a previously prepared pool of plasmid DNA from 43 different cloned 16S rRNA genes from deep-sea vent organisms (Huse *et al.*, 2007; Huber *et al.*, 2009).

We generated amplicon libraries using primer pools designed to span the V6 or V4-V5 hypervariable regions of as many known bacteria or archaea as possible (Tables S5 and S6). We used Invitrogen Platinum HiFi *Taq* polymerase for amplification, as we have found that the fidelity of standard *Taq* is insufficient for the level of deep sequencing provided by the GS FLX. We sequenced from the A adapter on a Roche GS FLX using standard Roche protocols and supplies and the amplicon base-calling pipeline. Sequences are available at the NCBI Short Read Archive (SRP001610).

Data from environmental samples were collected as previously described (Sogin *et al.*, 2006; Huber *et al.*, 2007; Gilbert *et al.*, 2009; Turnbaugh *et al.*, 2009; McLellan *et al.*, 2010) using the amplification and processing methods outlined above.

### Filtering low-quality and contaminating reads

For all data sets, we removed all sequences that contained one or more ambiguous bases (Ns), that did not have an exact match to the expected bar-coded forward primers, or that had an average quality score less than 30 (the V6 region is short and generally low in homopolymer stretches and therefore has high average quality scores) (Sogin *et al.*, 2006; Huse *et al.*, 2007; Kunin *et al.*, 2010). For V6 data sets, we also removed sequences that did not have a recognizable reverse primer sequence.

For non-environmental data sets, we compared all reads to a database of 16S rRNA sequences using GAST (Huse *et al.*, 2008). Reads that had a best match to a non-target sequence that was at least 10% better than the match to the nearest template sequence were considered to be contamination and were removed. Reads that either did not have any match or did not have a match over at least 80% of their length were considered to represent non-target amplification, chimeras or reads with gross errors and were removed. These sequences were compared with the GenBank nt database using BLASTN (Altschul *et al.*, 1990).

The likelihood of generating chimeras between short, hypervariable rRNA sequences of divergent taxa in the absence of the conserved regions of the gene is very small. The *E. coli* and *S. epidermidis* data sets, however, each include two very similar sequences in high density. Chimeras here are very similar to the correct sequences and map to the same species; therefore they are not identified by the minimum blast alignment requirement nor by standard chimera checking software, and would artificially increase the calculated error rate of PCR+pyrosequencing. Through visual examination we identified obvious chimeras and removed those specific sequences from the data. Some additional chimeras that contain sequencing errors and therefore do not exactly match predicted chimeras likely remain.

### Calculating the PCR+pyrosequencing error rate

We compared each read to the relevant set of template sequences using the needledist module of ESPRIT (with options −g 5.75 −e 2.75 −x) (Sun *et al.*, 2009). The error rate was calculated as the number of insertions and deletions (of any length) plus the number of individual substitutions divided by the length of the template sequence.

### Clustering reads into OTUs

We used mothur (Schloss *et al.*, 2009) to create a set of unique sequences from a fasta file of all quality-filtered reads in each sample. For unstructured multiple sequence alignments we used MUSCLE (Edgar, 2004) with non-default parameters −maxiters 2, −diags which have been previously shown to minimize alignment expansion in short hypervariable tags (Sogin *et al.*, 2006; Sun *et al.*, 2009). For Needleman-Wunsch pairwise alignments we used the kmerdist and needledist modules of ESPRIT (default parameters). We used quickdist (Sogin *et al.*, 2006) to create the distance matrix of all pairwise combinations from the multiple sequence alignment. ESPRIT's needledist creates a distance matrix using the quickdist distance calculation: the number of substitutions, deletions and insertions divided by the alignment length. We then used mothur to create OTUs using the single-linkage, average-linkage or complete-linkage options, and to calculate richness and rank-abundance statistics.

### Single-linkage preclustering

Our preclustering algorithm first orders the unique sequences by frequency, then steps through the ordered sequences, assigning them to clusters. The most abundant sequence starts the first cluster. Each subsequent sequence is tested against the growing list of clusters using the single-linkage algorithm. If the sequence has a pairwise distance less than 0.02 (equivalent to a single difference in the V6 region) to any of the sequences already in the cluster, the new sequence will be added to the cluster and not tested against subsequent clusters. If the sequence is not within a distance of 0.02 from any read in any of the existing clusters, it will establish a new cluster. Once all sequences have been assigned to clusters, sequences in the low abundance clusters (< 10 tags) are tested against the larger clusters and added to those clusters if possible. Ordering sequences by frequency ensures that those sequences that accurately represent the template pool will preferentially seed the establishment of a new cluster rather than those errant sequences that occur at lower frequency. This also helps avoid linking very different template sequences into a single cluster through a chain of low frequency errant sequences. The second round of preclustering incorporates small clusters into larger clusters in cases where the smaller cluster formed before the larger cluster included sequences that differ from the smaller cluster by less than 2%. For each final precluster, SLP assigns the sequence with the highest frequency to represent the cluster in downstream OTU clustering, with the count of all tags in the precluster assigned to it. The perl script that performs these operations, SLP, can be downloaded from http://vamps.mbl.edu/resources/software.php.

### PyroNoise comparisons

We randomly selected 10 000 tags having an average quality score ≥ 30 and no ambiguous bases. We ran PyroNoise with default values (including −s 15.0 −c 0.05 for PCluster) and removed denoised sequences that did not map to Bacteria by GAST. The remaining sequences were clustered using pairwise alignments and average-linkage clustering as above.

## Supporting information

**Fig. S1.** Reads with errors: observed vs. expected. We plotted the number of reads with one or more errors that we observed and that would be expected using a binomial distribution of errors. The binomial distribution underestimates the number of reads with multiple errors. The error rates used for each community are reported in Supplementary Table 2.

**Table S1.** Sequences generated from each clone experiment.

**Table S2.** Pyrosequencing and PCR error.

**Table S3.** PyroNoise comparison.

**Table S4.** Comparison of clustering methods after single-linkage preclustering (SLP).

**Table S5.** Amplification primers.

**Table S6.** Multiplexing barcodes used to differentiate subsamples of the two genomic template samples.
