# Supplementary figures and images for: Ironing out the wrinkles in the rare biosphere through improved OTU clustering
Source: Environ Microbiol. 2010 Jul;12(7):1889–98. doi: 10.1111/j.1462-2920.2010.02193.x (PMC2909393; doi:10.1111/j.1462-2920.2010.02193.x)

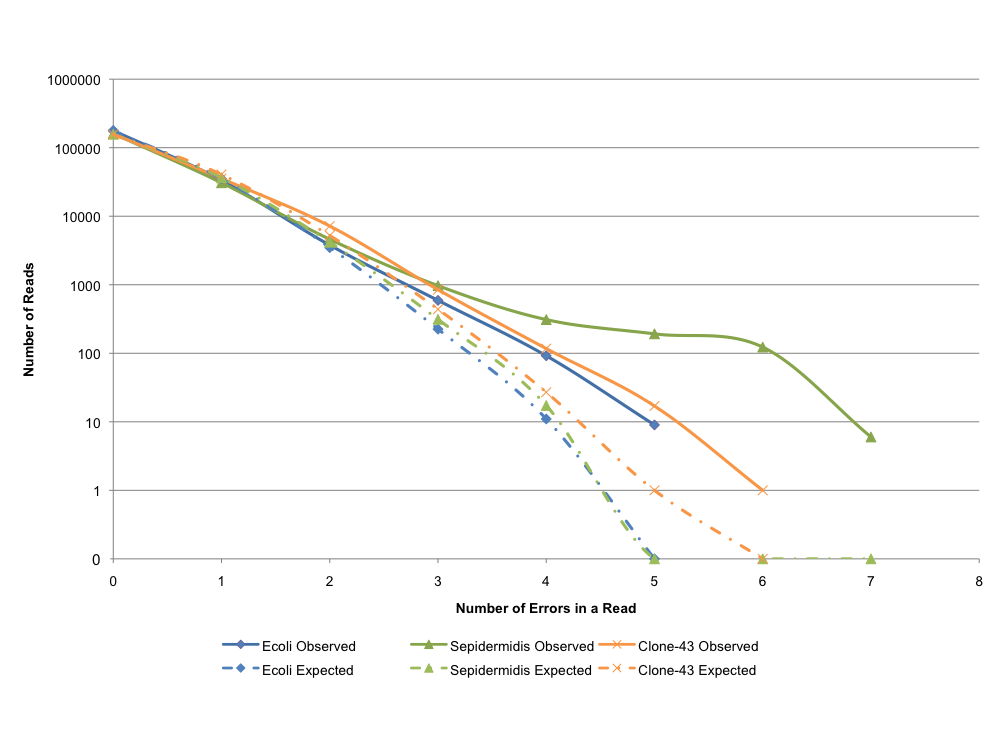

Supplement: Supplementary file 1 [file emi0012-1889-SD1.tif]
