# Supplementary material for: Ironing out the wrinkles in the rare biosphere through improved OTU clustering
Source: Environ Microbiol. 2010 Jul;12(7):1889–98. doi: 10.1111/j.1462-2920.2010.02193.x (PMC2909393; doi:10.1111/j.1462-2920.2010.02193.x)
Supplement: Supplementary file 2 [file emi0012-1889-SD2.doc]

Supplementary Table 1: Sequences generated from each clone experiment

The number of reads sequenced for each sample and the number of reads that were removed by each step of the quality and contamination filtering process.

|  | *E. coli* | *S. epidermidis* | *E. coli*  A operon | *S. epidermidis*  09 operon | Clone 43 |
| --- | --- | --- | --- | --- | --- |
| Total reads | 258,199 | 241,298 | 34,806 | 45,328 | 251,562 |
| High quality reads | 215,748 | 198,884 | 31,039 | 38,780 | 202,715 |
| Reads with BLAST …alignment <80% | 44 | 67 | 6 | 7 | 360 |
| Contaminating or …chimeric reads | 90 | 178 | 9 | 2 | 97 |
| Contaminating  …*E. coli* reads | - | 22 |  | 1137 | 49 |
| Reads used for …clustering | 215,614  (84%) | 198,617  (82%) | 31,024  (89%) | 37,634  (83%) | 202,209  (80%) |

Supplementary Table 2: Pyrosequencing and PCR error.

For each of 5 known template preparations, we calculated the number of sequences with zero, one, two or three errors and the number with greater than three errors. Estimates are based on pairwise alignment with the nearest template sequence.

|  | *E. coli* | *S. epidermidis* | *E. coli*  A operon | *S. epidermidis*  09 operon | Clone 43 |
| --- | --- | --- | --- | --- | --- |
| Errors per …base | 0.0033 | 0.0037 | 0.0021 | 0.0026 | 0.0042 |
| Reads with 0 …errors | 177,697  (82%) | 160,940  (81%) | 27,528  (89%) | 32,398  (86%) | 158,744  (78%) |
| Reads with 1 …error | 33,445  (16%) | 30,729  (16%) | 3,186  (10%) | 4,467  (12%) | 35,473  (18%) |
| Reads with 2 …errors | 3,784  (2%) | 4,607  (2%) | 286  (1%) | 618  (2%) | 7,183  (4%) |
| Reads with 3 …errors | 591  (<1%) | 969  (<1%) | 24  (<1%) | 78  (<1%) | 847  (<1%) |
| Reads with > …3 errors | 101  (<0.1%) | 631  (<1%) | 5  (<0.1%) | 26  (<0.1%) | 135  (<0.1%) |

Supplementary Table 3: PyroNoise comparison.

We estimated the additional OTUs predicted due to errors as 1-2 OTUs per 1,000 tags.

|  | *Additional predicted due to errors* | *MS-CL* | *SLP / PW-AL* | *PyroNoise/ PW-AL* |
| --- | --- | --- | --- | --- |
| Clone-43  (n=10,000) | 10-20 | 288 | 66 | 52 |
| Clone-90  (241nt, n=34,308) | 34-68 | 237 | 42 | 32 |
| Priest Pot (449nt, n=16,222) | 16-32 | 1188 | 394 | 656 |
| Deep-sea vent Archaea  (n=10,000) | 10-20 | 291 | 209 | 193 |
| English Channel  (n=10,000) | 10-20 | 756 | 593 | 686 |

Supplementary Table 4: Comparison of clustering methods after single-linkage preclustering (SLP).

|  | *SLP/ MS-CL* | *SLP/*  *PW-AL* |
| --- | --- | --- |
| *Template Samples* |  |  |
| *E. coli*  (n=215,618) | 88 | 88 |
| *E. coli* SSU A  (n=31,030) | 10 | 10 |
| *S. epidermidis*  (n=197,876) | 123 | 123 |
| *S. epidermidis SSU 9*  (n=37,587) | 20 | 20 |
| Clone-43 (v6)  (n=202,340) | 252 | 229 |
| Natural Samples |  |  |
| Deep-sea vent Archaea  (n=63,133) | 447 | 432 |
| English Channel  (n=12,851) | 810 | 788 |
| Human Gut  (n=15,239) | 555 | 511 |
| Sewage  (n=33,082) | 1739 | 1704 |
| North Atlantic Deep Water  (n=15,497) | 1292 | 1254 |

Supplementary Table 5: Amplification primers.

| Bacterial V6 967F pool | 5' CAACGCGAAGAACCTTACC  5' CTAACCGANGAACCTYACC  5' CNACGCGAAGAACCTTANC  5' CAACGCGAAAAACCTTACC 5' CAACGCGCAGAACCTTACC 5’ ATACGCGARGAACCTTACC |
| --- | --- |
| Bacterial V6 1064R pool | 5' CGACAGCCATGCANCACCT 5' CGACGGCCATGCANCACCT 5' CGACGACCATGCANCACCT 5' CGACAACCATGCANCACCT |
| Archaeal V6 958F | 5’ AATTGGANTCAACGCCGG |
| Archaeal V6 1064R | 5’ CGRCRGCCATGYACCWC |
| Bacterial V4-5 518F pool | 5’ CCAGCAGCCGCGGTAAN  5’ CCAGCAGCTGCGGTAAN |
| Bacterial V5-5 926R pool | 5’ CCGTCAATTCNTTTRAGT  5’ CCGTCAATTCTTTTGAGT  5’ CCGTCAATTTCTTTGAGT |

Supplementary Table 6: Multiplexing barcodes used to differentiate subsamples of the two genomic template samples.

| Sample Name | Library | Barcode Key |
| --- | --- | --- |
| *E. coli* Flask 1, PCR 1 | K12_1_1 | TGCAC |
| *E. coli* Flask 1, PCR 2 | K12_1_2 | GTATC |
| *E. coli* Flask 1, PCR 3 | K12_1_3 | GCTAC |
| *E. coli* Flask 2, PCR 1 | K12_2_1 | ACGCA |
| *E. coli* Flask 2, PCR 2 | K12_2_2 | GAGAC |
| *E. coli* Flask 2, PCR 3 | K12_2_3 | GACTC |
| *E. coli* Flask 3, PCR 1 | K12_3_1 | CTAGC |
| *E. coli* Flask 3, PCR 2 | K12_3_2 | CGCTC |
| *E. coli* Flask 3, PCR 3, emPCR 1 | K12_3_3 | TGATA |
| *E. coli* Flask 3, PCR 3, emPCR 2 | K12_3_4 | TGATA |
| *E. coli* single plasmid clone | K12_CLN_D04 | CGCAG |
| *S. epidermidis* Flask 1, PCR 1 | Staph_1_1 | TCACA |
| *S. epidermidis* Flask 1, PCR 2 | Staph_1_2 | TAGCA |
| *S. epidermidis* Flask 1, PCR 3 | Staph_1_3 | ACTAT |
| *S. epidermidis* Flask 2, PCR 1 | Staph_2_1 | CAGTA |
| *S. epidermidis* Flask 2, PCR 2 | Staph_2_2 | ATCGA |
| *S. epidermidis* Flask 2, PCR 3 | Staph_2_3 | ATATA |
| *S. epidermidis* Flask 3, PCR 1 | Staph_3_1 | AGACA |
| *S. epidermidis* Flask 3, PCR 2 | Staph_3_2 | ACAGA |
| *S. epidermidis* Flask 3, PCR 3, emPCR 1 | Staph_3_3 | TGTGC |
| *S. epidermidis* Flask 3, PCR 3, emPCR 2 | Staph_3_4 | TGTGC |
| *S. epidermidis* single plasmid clone | Staph_CLN_E03 | GCGAT |
